# Soluble and Cell–Cell-Mediated Drivers of Proteasome Inhibitor Resistance in Multiple Myeloma

**DOI:** 10.3389/fendo.2018.00218

**Published:** 2018-05-01

**Authors:** Mariah L. Farrell, Michaela R. Reagan

**Affiliations:** ^1^Reagan Laboratory, Maine Medical Center Research Institute, Scarborough, ME, United States; ^2^Graduate School of Biomedical Sciences and Engineering, University of Maine, Orono, ME, United States; ^3^School of Medicine, Tufts University, Boston, MA, United States; ^4^Sackler School of Graduate Biomedical Sciences, Tufts University, Boston, MA, United States

**Keywords:** multiple myeloma, drug resistance, bone marrow MSCs, bortezomib, carfilzomib, ixazomib

## Abstract

It is becoming clear that myeloma cell-induced disruption of the highly organized bone marrow components (both cellular and extracellular) results in destruction of the marrow and support for multiple myeloma (MM) cell proliferation, survival, migration, and drug resistance. Since the first phase I clinical trial on bortezomib was published 15 years ago, proteasome inhibitors (PIs) have become increasingly common for treatment of MM and are currently an essential part of any anti-myeloma combination therapy. PIs, either the first generation (bortezomib), second generation (carfilzomib) or oral agent (ixazomib), all take advantage of the heavy reliance of myeloma cells on the 26S proteasome for their degradation of excessive or misfolded proteins. Inhibiting the proteasome can create a crisis specifically for myeloma cells due to their rapid production of immunoglobulins. PIs have relatively few side effects and can be very effective, especially in combination therapy. If PI resistance can be overcome, these drugs may prove even more useful to a greater range of patients. Both soluble and insoluble (contact mediated) signals drive PI-resistance *via* activation of various intracellular signaling pathways. This review discusses the currently known mechanisms of non-autonomous (microenvironment dependent) mechanisms of PI resistance in myeloma cells. We also introduce briefly cell-autonomous and stress-mediated mechanisms of PI resistance. Our goal is to help researchers design better ways to study and overcome PI resistance, to ultimately design better combination therapies.

## Myeloma and Proteasome Inhibitors (PIs)

In 2017, there were an estimated 30,280 new cases of multiple myeloma (MM) diagnosed and ~12,590 deaths due to MM, which comprised ~2% of all cancer deaths (1). Although myeloma is typically considered an incurable cancer of the plasma cell, the overall survival for myeloma patients has improved from a prior median of 2.75 years around 1998 (1), to 6 years in 2010 (2), and up to 7.7 years for patients under 65 years old diagnosed between 2008 and 2015 (3). Current advances in the field aim to develop novel therapies using new targets in myeloma, determine better biomarkers for response or progression from the precursor disease monoclonal gammopathy of undefined significance, develop better combination treatments, and understand how to overcome drug resistance that occurs due to mutations or effects of the microenvironment on myeloma cells.

The proteasome is a multi-enzyme complex of the ubiquitin–proteasome system, which governs destruction of unwanted intracellular proteins and is needed to retain cellular health and homeostasis (3). Inhibition of proteasomal degradation results in cell apoptosis and death. Clinically, PIs are very useful in myeloma and other cancers. Bortezomib, a peptide boronic acid, is a slowly reversible inhibitor of the β5 catalytic subunit within the 20S catalytic core complex. Carfilzomib irreversibly inhibits the same β5 site. Ixazomib is similar to bortezomib, and oprozomib is similar to carfilzomib, but both are, conveniently, orally administered. The investigational agent marizomib has a β-lactone unit that results in irreversible inhibition of both β2 and β5 catalytic sites (1). Off-target effects of PIs are typically minimal and can potentially be overcome with oral versions or tumor- or bone-targeted nanomedicine delivery systems (4). Gastrointestinal and cardiovascular toxicities, and other toxicities such as rash, have been observed with PIs (1).

**Figure 1:**
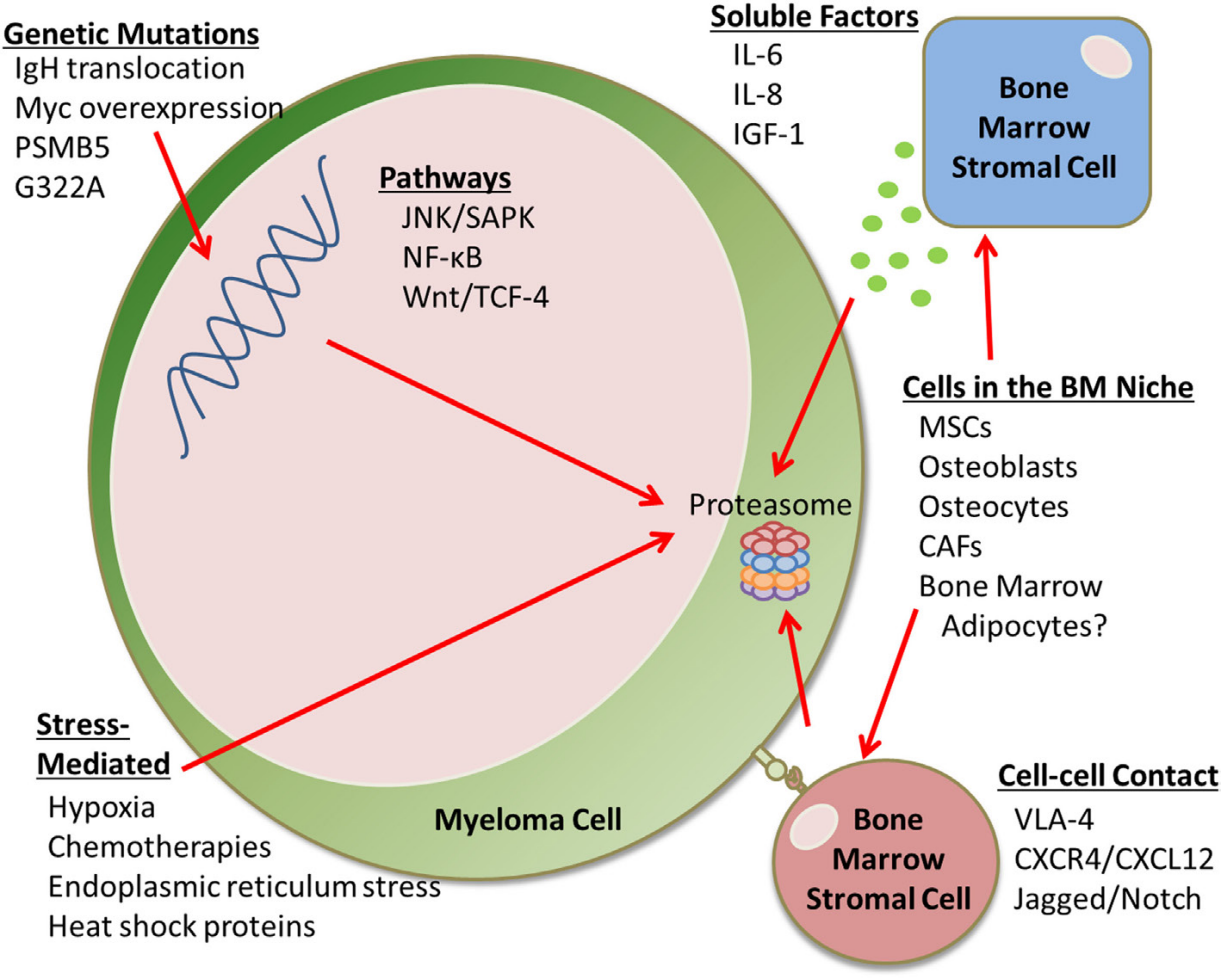
Proteasome inhibition resistance mechanisms. This mini-review discusses the many factors that contribute to proteasome inhibitor (PI) resistance in the bone marrow (BM). For example, there are genetic mutations that can lead to drug resistance, as well as soluble factors and cell–cell contact-mediated signals from an array of BM stromal cells that can cause PI resistance. Cells that can cause drug resistance include mesenchymal stem cells (MSCs), osteoblasts, osteocytes, cancer-associated fibroblasts (CAFs), and potentially BM adipocytes. Stress-mediated responses can also cause PI resistance.

Proteasome inhibitors inhibit key autocrine and paracrine signaling intracellular pathways associated with myeloma cell growth and survival, often signaled by extracellular matrix and cells of the bone marrow (BM), such as mesenchymal stromal cells (MSCs). PIs suppress the production of cytokines including interleukin-6 (IL-6), insulin-like growth factor 1 (IGF-1), and tumor necrosis factor α (TNFα), which can affect MSC and myeloma cell interactions (5). Interestingly, PIs can also suppress angiogenesis by decreasing VEGF secretion (5). PIs allow for the accumulation of misfolded and unfolded proteins, resulting in endoplasmic reticulum (ER) stress, reactive oxygen species (ROS)-induced oxidative stress, and the unfolded protein response in myeloma cells. PIs also inhibit NF-κB signaling (3), a major growth and survival signaling pathway in MM, which was the original reason for pursuing PIs in MM. However, NF-κB inhibition alone cannot account for the overall anti-MM activity of bortezomib, as demonstrated by studies comparing bortezomib to the IKK-B-specific inhibitor PS1145 (3). PIs also upregulate p53, a tumor suppressor that upregulates p21^Waf1^ to induce cell cycle arrest (5). PIs can induce apoptosis through extrinsic caspase-8 cascade *via* activation of JNK, and *via* caspase-9 cleavage, associated with the upregulation of Noxa and inhibition of antiapoptotic Bcl-2 and XIAP family proteins (5, 6). PIs also suppress adhesion molecule and growth factor receptor expression (e.g., IL-6R) and inhibit cellular mechanisms for repairing double-strand DNA breaks (7). Unfortunately, many patients develop PI-refractory MM; the mechanisms of this resistance is discussed here (Figure 1).

## Stress-Mediated Responses

Bortezomib can inhibit chymotrypsin-like proteasome activity in both bortezomib-sensitive and bortezomib-resistant cell lines, demonstrating that certain forms of bortezomib resistance are not dependent on the type or extent of proteasome inhibition (8). This suggests that certain pathways, such as stress-related pathways, are altered in PI-resistant cells, which may change their dependency on proteasome activity. Hypoxia, a state of low oxygen tension, can result from rapid tumor growth or be induced by chemotherapy. Muz and colleagues found that hypoxia drives PI resistance in MM1S, OPM1, and H929 myeloma cells (9). Raninga et al. also found that hypoxic conditions induced bortezomib resistance; this resistance was linked to a decrease in NF-κB regulated genes (10). Treatment with selinexor, the first drug in a new class of agents known as Selective Inhibitor of Nuclear Export (SINE™) compounds, overcame hypoxia-induced bortezomib resistance by targeting the nuclear export protein exportin 1 (XPO1) in MM cells (11). Selinexor combined with bortezomib decreased tumor burden and extended survival in mice inoculated with bortezomib-resistant MM1S (11). Thus, selinexor and other inhibitors of XPO1, a protein found in the nucleus of cancer cells, hold great promise for combination therapy with PIs; currently, the STORM, STOMP, and BOSTON clinical trials are exploring this avenue.

Heat shock proteins (HSPs) are chaperone proteins that play a significant role in stressful conditions, such as chemotherapy exposure, and especially upon ER stress, typically triggered by accumulation of unfolded proteins. Many HSP-related genes are overexpressed, including HSP70, in bortezomib-resistant cells (8). Hamouda et al. demonstrated that HSPB8 gain or loss of function was a key factor in bortezomib resistance in U266 myeloma cells (12). Hsp27 has also been linked to bortezomib resistance, and Yasui et al. were able to overcome this by co-treating with BIRB 796 (13). In the study, bortezomib triggered upregulation of p38/MAPK and phosphorylation of Hsp27; BIRB 796 blocked this from occurring and ultimately led to cell death (13). Similarly, inhibiting Hsp90 with KW-2478, and co-treating with bortezomib induced caspase activation *in vitro*, and synergistic antitumor activity *in vivo* (14). Furthermore, Shringarpure et al. demonstrated that HSPs (HSP27, HSP70, and HSP90) and other chaperone proteins were more highly expressed in bortezomib-resistant SUDHL-4 lymphoma cells than in bortezomib-sensitive cells (8). HSP27 expression was also elevated in bortezomib-resistant HT-29 adenocarcinoma cells (15). Overall, the upregulation stress response genes and proteins, which cause cell survival and induce antiapoptotic pathways, induce PI resistance in many tumor types. For more on ER stress roles in the development of MM and drug resistance, we refer the reader to the recent review from Nikesitch et al. (16).

Environmental stresses, inflammatory cytokines, growth factors, and GPCR agonists can all also activate the JNK/SAPK pathway in myeloma cells. However, the role of this pathway in bortezomib is controversial. Some groups have found that bortezomib increases the stress kinase JNK pathway to induce apoptosis in myeloma cells (17) or cell death by overproduction of mitochondrial ROS (18); others suggest that the JNK signaling in myeloma cells induces their proliferation and PI-resistance (19, 20). The complicated feedback and overlap between the intercellular cellular signaling pathways further complicates identifying the pathways through which MM cells overcome PIs.

Recently, bortezomib has been shown to interfere with general protein biosynthesis at the stage of nuclear ribosome biogenesis (21). Galimberti et al. found that bortezomib-induced changes in cytoplasm morphology and nucleolar ultrastructure. These changes were associated with the accumulation of transcription factor (TF) ATF4 at nucleolar sites in ovarian and cervical cancer cells (21). ATF4 is a stress-inducible TF and it accumulates at specific rRNA-processing nucleolar regions. Thus, increased expression of proteins in this family may allow cells to survive under conditions of high proteotoxic cell stress and these proteins may be used by PI-resistant cells to handle stress induced by bortezomib. In lymphoma cells, ATF3, ATF4, and ATF5 can be induced by bortezomib treatment, but confusingly, their overexpression is associated with bortezomib sensitivity (8). Inhibiting the AAA ATPase, p97 with CB-5083 has recently shown excellent potential for overcoming PI resistance induced by p97-dependent retro-translocation of the TF, Nrf1, which transcribes proteasome subunit genes following exposure to a PI (22). More research into these TFs and epigenetic modulators in myeloma PI resistance is warranted.

Metabolic changes in MM cells have recently been discovered to contribute to PI-resistance. Cellular bioenergetics is significantly different between PI-resistant cells and their sensitive counterparts. Recent work from Thompson et al. demonstrated that targeting glutamine-induced respiration in PI resistant cells, using the glutaminase-1 inhibitor CB-839, synergized with PIs to induce cytotoxicity in MM cells (22). Targeting cellular metabolic pathways, and understanding how BM components change MM cell metabolic pathways, is likely an untapped resource in the fight against drug resistance in MM.

## Genetic Mutation-Mediated Drug Resistance

In myeloma (23) and mesothelioma (24), high basal levels of proteasome activity or upregulation of proteasome subunits can overcome PI treatments. This finding suggests that an unfavorable load-versus-capacity balance represents a critical determinant of primary apoptotic sensitivity to bortezomib; understanding what modulates proteasomal activity thus may help in overcoming resistance. Genetic mutations, including mutations in the proteasome subunit 5 (PSMB5), mutations causing overexpression of MYC, and translocations of IgH gene locus, can induce very high basal levels of proteasome activity that overwhelm effects of proteasome inhibition. Oerlemans and colleagues observed that PI-resistant THP1 cells had a 60-fold increase in protein levels for PSMB5 and a G322A point mutation in the PSMB5 β-subunit of the bortezomib-binding pocket (25). A similar discovery was made by Balsas et al. in RPMI8226 myeloma cells, where overexpression of PSMB5 at the mRNA and protein level (although without a G322A mutation) was linked to their bortezomib resistance. Co-treatment with the histone deacetylase (HDAC) inhibitor trichostatin A induced synergistic effects with bortezomib to induce apoptosis (26). Indeed, HDAC inhibitors show efficacy in many combinatorial therapies to kill PI-resistant cells (27). Others have confirmed upregulation of PSMB5 gene and G332A mutations in other PI-resistant MM cells, which have a mutation cluster region in the binding pocket, particularly the S1 specificity pocket (28, 29). PSMB5 mutations had not been identified in humans until recently when Barrio et al. found certain subclones, resulting from branching evolution, that had mutations within PSMB5 resulting in PI resistance (30).

## Soluble Factor-Mediated Drug Resistance

Many soluble factors, including IL-6, IL-8, and IGF-1, in the BM microenvironment can also contribute to PI resistance. Voorhees and colleagues specifically found that CNTO 328, a monoclonal antibody against IL-6, enhanced cytotoxicity of bortezomib, activated caspase-3, -8, and -9, and induced HSP70 (31). Similarly, BM cancer-associated fibroblasts (CAFs) have recently been shown to protect against PI-induced apoptosis in myeloma cells, and produce high levels of IL-6, IL-8, and TGFβ (23). Bortezomib was found to induce ROS and autophagy in bortezomib-resistant CAFs by inhibiting mTOR and p62 and increasing light chain 3 protein-II (32). TGFβ was found to mediate bortezomib-induced autophagy, and a combination of bortezomib plus LY2109761, a selective TGFβRI/II inhibitor, induced apoptosis of RPMI8226 myeloma cells co-cultured with bortezomib-resistant CAFs (32). This study demonstrates how targeting stroma cells and stroma-derived factors can be useful in overcoming drug resistance and exemplifies how myeloma cells hijack their microenvironment to make it more tumor-supportive.

Similarly, primary MM cells have been found to be resistant to bortezomib partially from BM-MSC-derived cytokines. Interestingly, bortezomib and IL-8 may be involved in a positive feedback loop: in a study in which BM-MSCs were extracted from myeloma patients, bortezomib-resistant tumor cells had increased activity in the NF-κB pathway due to release of IL-8 from the MSCs (33). Myeloma patient MSCs secreted more IL-8 than healthy MSCs, and this was mimicked with cell co-cultures *in vitro*. Bortezomib further increased IL-8 expression from osteoclasts, stromal cells, and myeloma cell lines (23). Bortezomib may increase the expression of IL-8 through the p38 MAPK pathway (34). Kuhn et al. found that blocking IGF-1 or IGF-1R increased myeloma cell death synergistically when co-treating with bortezomib in cell lines and patient samples (35). Zheng et al. also found that mTOR and ERK1/2 signaling, *via* thioredoxin, can induce PI-resistance (36). The Azab lab also found that PI3K signaling in myeloma cells, and the PI3K-α isoform specifically, was induced by co-culturing myeloma cells with BM-MSCs and induced bortezomib resistance (37). The Ghobrial lab similarly found that a pan-class I PI3K inhibitor, buparlisib, could reduce MSC-induced survival in myeloma cells (38). These data suggest that PI3K and mTOR pathways contributed to PI resistance.

In addition to PI3K, NF-κB, activated *via* a range of stimuli, including ROS, TNFα, and IL-1β, is another pathway through which MSCs induce bortezomib resistance (33). MSC-derived exosomes induced PI-resistance in myeloma cells and contained contents that modulated JNK, p38, p53, and Akt pathways in myeloma cells (39). The HS-5 cell line has also been shown to induce bortezomib resistance in myeloma cells through CK2, a pivotal pro-survival kinase that activates NF-κB and STAT3 (40). The NF-κB signaling pathway has been shown to play a role in PI-resistance through numerous downstream signals including the upregulation of antiapoptotic BCL-XL (41). Moreover, hyaluronan and proteoglycan link protein 1 is produced in BM stromal cells from MM patients, is detected in patients’ BM plasma, and has been shown to activate an atypical bortezomib-resistant NF-κB pathway in MM cells (16). Extensive new research has confirmed NF-κB signaling as critical in bortezomib resistance in MM cells (16). B-cell activating factor (BAFF), a cytokine in the TNF ligand family, is another important molecule shown to induce PI-resistance in MM cells (42). BAFF can drive macrophage-mediated PI-resistance and suppress caspase activation in MM cells through activation of Src, Erk1/2, Akt, and NF-κB signaling (42). Recent work from Qin et al. has shown that anti-BAFF-R antibody therapies had remarkable single-agent antitumor effects and induced potent antibody-dependent cellular cytotoxicity (ADCC) against multiple subtypes of human lymphoma and leukemia (16); we propose these may be useful in MM as well.

Macrophage inflammatory protein-1α is another macrophage (and myeloma) cell-derived cytokine that is able to induce bortezomib resistance. It functions through activation of ERK1/2, Akt, and mTOR pathways (43). Que et al. showed that the receptor tyrosine kinase c-Met is overexpressed in human myeloma cell lines and also causes PI-resistance *via* increased Akt/mTOR signaling (44). Akt can be activated by numerous agents (cytokines, integrins, RTKs, BCR signaling, and GPCR ligands) and can be downstream of the Jak1 and PI3K pathways. Recent studies in myeloma cells with N- and K-Ras mutations suggest that aspirin can increase the efficacy of bortezomib treatment *via* suppression of Akt phosphorylation, upregulation of survivin, and in part through suppressing Bcl-2 levels (45). The allosteric AKT inhibitor MK2206 was also found in myeloma cells to overcome bortezomib resistance induced by IL-6 or MSCs (46). Akt signaling has also been tied to autophagy, which is another mechanism of bortezomib resistance. Autophagy can result from signaling through CLCN5 (a member of the chloride channel family), which functions through the AKT/mTOR pathway (47). Blocking the mTOR/PI3K and Rad (Ras associated with diabetes) pathways have also been shown to overcome PI-resistance in lymphoma and hold potential in myeloma (48).

Soluble factors can also activate the Wnt signaling pathway, and the Wnt/TCF-4 signaling pathway may also be involved in PI resistance. In a bortezomib-resistant lymphoma cell line, increased TCF4 expression and increased transcription by the TCF-4/β-catenin complex was observed, accompanied by upregulation of their downstream target genes (c-myc and cyclin D1) (8). In myeloma, the β-catenin inhibitors BC2059 (46) and polyphyllin I (49) have been shown to be efficacious in combination with bortezomib.

## Cell–Cell Contact-Mediated Drug Resistance

The BM niche contains many cells that directly interact with and alter myeloma cells in a bidirectional manner, leading to changes in both that support tumor progression, osteolysis, and disrupted hematopoiesis (50–53). Cell–cell contact-mediated drug resistance has become a widely recognized mechanism of drug resistance in the BM. Specific adhesion molecules of interest for MM PI-resistance include very late antigen-4 (VLA-4), CXC chemokine 12 (CXCL12) and its receptor CXC chemokine receptor 4 (CXCR4), and Jagged/Notch. VLA-4 has been linked to cell adhesion-mediated drug resistance (CAM-DR), and Noborio-Hatano et al. identified the α4-integrin (a subunit of VLA-4) as responsible for multiple drug resistance in myeloma (54). BM-MSCs cause CAM-DR in part through the CXCR4/CXCL12 axis. Waldschmidt et al. showed that inhibiting CXCR4 with plerixafor or CXCL12 with NOX-A12 resensitized MM to PIs (55).

Jagged-1/Notch signaling has also been associated with PI resistance in MM, and this has been shown to be overcome with the use of PKC inhibitors (56). When notch ligand Dll1 on BM stromal cells binds to Notch2 receptor on myeloma cells, a cascade results that upregulates a cytochrome P450 enzyme involved in drug metabolism (CYP1A1), which ultimately leads to PI resistance. Treatment of cells with α-Naphthoflavone or CYP1A1 siRNA reintroduced PI sensitivity in myeloma cells (57). In sum, Jagged-1/Notch signaling has been a major pathway of focus for drug resistance in MM cells.

DTX3L is an ubiquitin ligase that plays an essential role in cell cycle and promotes adhesion to BM stromal cells or fibronectin. In work by Liu et al., inhibiting DTX3L induced an apoptotic response to bortezomib in myeloma cells (58). DTX3L was found to be regulated by focal adhesion kinase and represents another pathway through which CAM-DR is induced in myeloma cells. Bustany et al. compared myeloma cells that continually express cyclin D1 versus parental controls. Similarly with DTX3L, cyclin D1 expression increased myeloma adhesion to stromal cells and fibronectin. These cells also had stabilized F-actin fibers, enhanced chemotaxis, and inflammatory chemokine secretion. Both parental and cyclin D1-expressing cells were resistant to acute carfilzomib treatment when cultured on stromal cells, but this could be overcome in cyclin D1-expressing cells after pretreatment with lenalidomide. The team found changes in myeloma cell metabolism (specifically, increases in ROS) in cyclin-D expressing cells, and resulting increases in oxidative stress-induced ERK1/2 signaling (59).

## Future Directions

There remains a great need to overcome bortezomib resistance in myeloma. As described here, AKT/PI3K and NF-κB pathways are heavily involved drug resistance in MM. Interestingly, there may be crosstalk between these two major pathways that has yet to be explored. Kloo et al. found that inhibiting PI3K in activated B cell like diffuse large B cell lymphoma (ABC DLBCL) cells decreased NF-κB target genes, which led to decreased survival of the ABC DLBCL cells (60). Similarly, findings in a study using an iMYC^Eµ^ B lymphoma line created by Han et al. suggested that constitutive activation of NF-κB and STAT3 was dependent on signaling through the PI3K pathway and was essential for survival and proliferation (61). While these studies did not look specifically at PI drug resistance, the crosstalk could be implicated in the drug resistance in myeloma. As this is becoming a growing focus in the lymphoma cancer field and because many labs have shown the importance of these two pathways in the progression of MM, this crosstalk should be investigated.

The future of PI-resistance, and drug resistance in general, will be greatly aided by advances in high-throughput “-omics” techniques that create an unprecedented opportunity for understanding PI-resistance at the genomic, transcriptomic, and proteomic level. Novel PIs are also being developed that can likely overcome resistance by targeting two or more proteasome subunits, such as the syringolin analog, syringolog-1, which inhibits the activity of both the β5 and β2 subunits (22).

As stemness, dedifferentiation, and drug resistance often correlate, a better characterization of the myeloma stem cell will likely provide even more information about drug resistance and emergence of a drug-resistant clone from a parental population (23, 62). Interestingly, Gu et al. demonstrated that inducing differentiation of MM cells made these more sensitive to bortezomib (23). For example, the blockade of PAX5 (also known as B cell-specific activator protein) (63) and changes in X-box-binding protein (62) TF splicing induce differentiation and targeting these proteins has been shown to reduce bortezomib resistance in MM (64). Finally, one of the areas that hold great potential for overcoming drug resistance is through therapeutically targeting BM stromal cells that have not previously been targeted, such as the BM adipocytes. As Falank et al. have recently shown, BM adipocytes may induce drug resistance in MM cells through both soluble and cell–cell contact-mediated mechanisms (65). More research into the roles of BM adipocytes in MM drug resistance is warranted.

## Conclusion

Proteasome inhibitor resistance occurs through a variety of mechanisms, which evade different functions of PIs. PIs can also synergize or have additive activity with other chemotherapies or myeloma-targeted agents, and PI-based combination regimens are ubiquitous in myeloma treatment algorithms for clinicians, which often comprise immunomodulatory drugs, monoclonal antibodies, and HDAC inhibitors. However, myeloma patients may be resistance to PIs, based on a certain mutation, epigenetic change, or microenvironmental influence on their tumor cells, and patients often become refractory to PIs due to emergence of a PI-resistant clone. It is likely that more mechanisms of PI-resistance exist and these should be further explored. Combination therapies have proven essential for overcoming PI resistance in myeloma and other cancers, and further research in this arena, especially with consideration as to how to target the stroma or overcome stroma-induced PI-resistance, will likely further improve treatment options for myeloma patients. For more reading on the 26S PI resistance in myeloma beyond this mini-review, we refer the reader to the reviews by Gandolfi et al. (1), Larocca et al. (66), and Ziogas et al. (67).

## Author Contributions

MF and MR co-wrote and co-edited this review.

## Conflict of Interest Statement

The authors declare that the research was conducted in the absence of any commercial or financial relationships that could be construed as a potential conflict of interest. The handling Editor declared a past co-authorship with the author MR.
